# Global surgery education in Europe: a landscape analysis

**DOI:** 10.1093/bjsopen/zrac001

**Published:** 2022-02-02

**Authors:** Lotta Velin, Adriana C. Panayi, Iris Lebbe, Emmanuelle Koehl, Gauthier Willemse, Dominique Vervoort

**Affiliations:** Centre for Teaching & Research in Disaster Medicine and Traumatology (KMC), Department of Biomedical and Clinical Sciences, Linköping University, Linköping, Sweden; Division of Plastic Surgery, Department of Surgery, Brigham and Women’s Hospital, Harvard Medical School, Boston, Massachusetts, USA; School of Clinical Medicine, University of Cambridge, Cambridge, UK; Faculty of Medicine, Katholieke Universiteit Leuven, Leuven, Belgium; Harvard T.H. Chan School of Public Health, Boston, Massachusetts, USA; Harvard T.H. Chan School of Public Health, Boston, Massachusetts, USA; Faculty of Medicine, Katholieke Universiteit Leuven, Leuven, Belgium; Institute of Health Policy, Management and Evaluation, University of Toronto, Toronto, Ontario, Canada

*Dear Editor*

Globally, there is a large unmet need for safe, timely and affordable surgical healthcare^1^. Since the 2015 Lancet Commission on Global Surgery, the movement has grown, including development of National Surgical, Obstetric and Anaesthesia Plans (NSOAPs)^2^ and increased demand for global surgery opportunities^3,4^; however, little is known about academic global surgery in Europe.

The authors performed an analysis of global surgery educational programmes and opportunities across Europe, which may serve as a reference for trainees and attendings interested in global surgery and benchmarks for budding programmes.

A list of medical schools in the European Economic Area, UK and Switzerland was obtained from the World Directory of Medical Schools^5^. For each institution, websites and grey literature were reviewed to determine the presence of: centres/initiatives in global surgery, official educational programmes in global surgery or in global health with a surgery track, and *ad hoc* academic global surgery opportunities (defined as activities not under the umbrella of a centre or educational programme; excluding student-only groups). All opportunities were reviewed, and the following variables extracted: year of onset, partner countries, nature of activities and main working language.

**Fig. 1:**
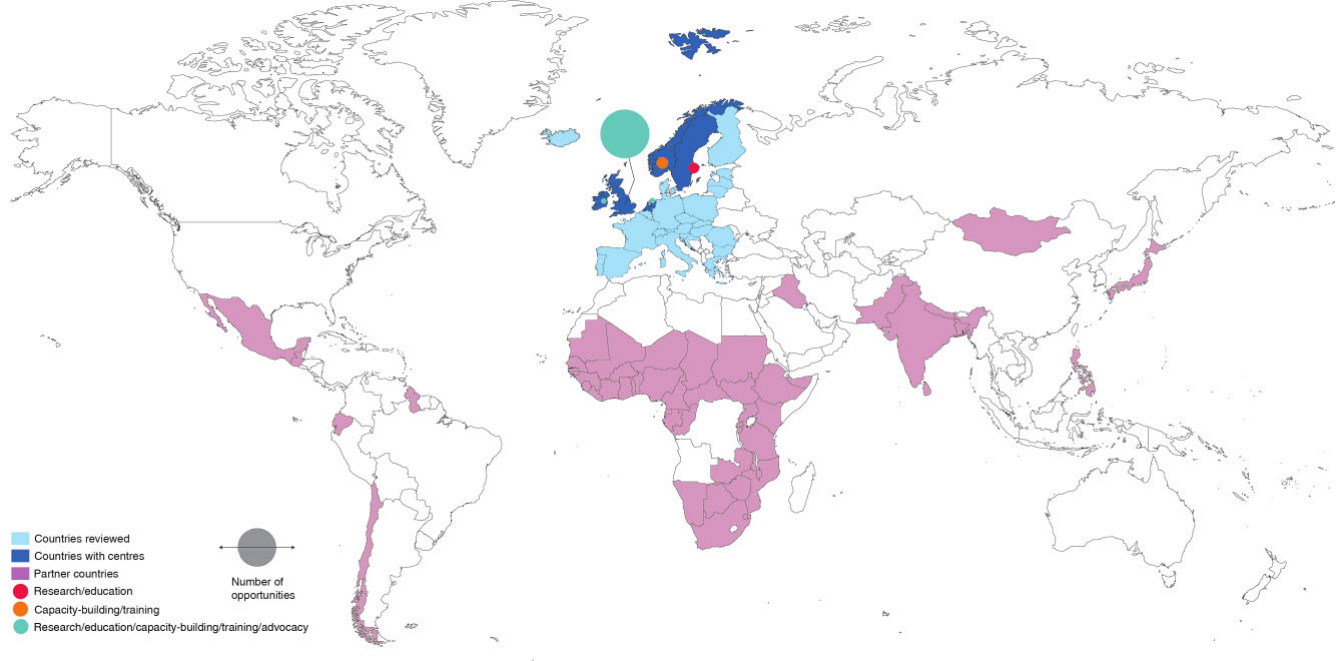
Global surgery centres in Europe by country, type of activities, and partner countries

The authors identified 347 medical schools across 31 countries in Europe. Sixteen (4.6 per cent) universities had a global surgery centre (*Fig. 1*); most (10 centres) were in the UK, followed by Sweden and Norway (2 centres each). Most (10 centres) worked primarily in English. Partner countries were listed by 10 centres, which included 55 countries, of which 40 (73 per cent) were in the African region, five (9 per cent) in the Pan-American region, and four (7 per cent) in the South-East Asian region. The most common partner countries were Sierra Leone (*n* = 7), India (*n* = 4), and Ethiopia (*n* = 4). Non-specific surgery centres or initiatives constituted five of the centres, whereas four were centres or initiatives working on specific global surgery issues, such as trauma/injury or burn injuries, and three were global health centres with a targeted global surgery group. The majority (12 centres) incorporated research in their work, seven had capacity-building components, and three had formal education opportunities (for example, electives or special study modules).

Five (1.4 per cent) universities had global surgery educational programmes, of which one had a global surgery elective, one had an elective in global surgery and paediatrics, one had a global health master programme with a surgery track, and one had an elective in humanitarian surgery. The Royal College of Surgeons in Ireland offered two programmes through the School of Medicine in Dublin: one Master programme in surgery with one module on ‘Surgery in the developing world,’ and one Master programme in Surgical Science and Practice with a global surgery submodule.

The authors identified *ad hoc* opportunities in 19 universities (5.5 per cent), of which 12 had structured research projects and seven had educational opportunities, such as summer schools, lecture series and conferences.

The findings suggest that academic global surgery opportunities remain relatively scarce in Europe. The authors propose expansion of global surgery education in Europe to meet the large interest among trainees and strengthen the role of European stakeholders in the global surgery discourse. It is unclear if the centres identified in this study had access to funding and limited information was available on the presence of faculty members and scholarships. Mobilizing increased funding might broaden opportunities for trainees from Europe and beyond.

*Disclosure*. The authors declare no conflict of interest.
